# Recognition of Mould Colony on Unhulled Paddy Based on Computer Vision using Conventional Machine-learning and Deep Learning Techniques

**DOI:** 10.1038/srep37994

**Published:** 2016-11-29

**Authors:** Ke Sun, Zhengjie Wang, Kang Tu, Shaojin Wang, Leiqing Pan

**Affiliations:** 1 College of Food Science and Technology, Nanjing Agricultural University, No. 1 Weigang Road, Nanjing 210095, P. R. China; 2 Department of Biological Systems Engineering, Washington State University, 208 L.J. Smith Hall, Pullman, WA 99164-6120, USA

## Abstract

To investigate the potential of conventional and deep learning techniques to recognize the species and distribution of mould in unhulled paddy, samples were inoculated and cultivated with five species of mould, and sample images were captured. The mould recognition methods were built using support vector machine (SVM), back-propagation neural network (BPNN), convolutional neural network (CNN), and deep belief network (DBN) models. An accuracy rate of 100% was achieved by using the DBN model to identify the mould species in the sample images based on selected colour-histogram parameters, followed by the SVM and BPNN models. A pitch segmentation recognition method combined with different classification models was developed to recognize the mould colony areas in the image. The accuracy rates of the SVM and CNN models for pitch classification were approximately 90% and were higher than those of the BPNN and DBN models. The CNN and DBN models showed quicker calculation speeds for recognizing all of the pitches segmented from a single sample image. Finally, an efficient uniform CNN pitch classification model for all five types of sample images was built. This work compares multiple classification models and provides feasible recognition methods for mouldy unhulled paddy recognition.

Rice is an important staple food worldwide, especially in Asia^1^. The total production of paddy (unmilled rice) globally was nearly 738 million tons in 2013, and the main product distribution regions are China, Brazil and America (FAOSTAT). Rice is usually stored as unhulled paddy after harvest. During storage, unhulled paddy can easily become infected with mould and lose its human-consumption value. Mycotoxin, a secondary metabolite of mould, may induce many types of disease, including cancers, in the human body^2^. *Aspergillus* and *Penicillum* are the main microorganisms that cause mould on paddy^3^. Due to the different toxin tolerances of animals, paddy may still have some feeding value for the pig and poultry industries after becoming slightly mouldy^4^. However, it can still cause health problems in animals if the type and amount of mycotoxin are out of control^5^. There are some methods to reduce the mycotoxin content in mouldy unhulled paddy, including removing the badly moulded paddy grains, which contain high levels of mycotoxin; mixing the mouldy unhulled paddy with a certain percentage of intact paddy^6^; and some chemical and biological mycotoxin reduction methods. Because the type and amount of mycotoxin can have a great influence on the safety of feedstuff made of mouldy unhulled paddy, recognition of the main infected mould species and mould colony distribution is crucial for enabling the utilization of mouldy paddy.

Computer vision, which simulates the human visual system, is a detection technology with the advantages of high detection speeds, low cost, easy maintenance and high visualization. Computer vision technology is often used for the rapid detection of agricultural product properties, such as quality detection for grains, vegetables and fruits^7^,^8^,^9^. Conventional linear and non-linear machine-learning models, such as the partial least squares (PLS) method, support vector machine (SVM) and artificial neural network (ANN), have played important roles in computer vision technology^10^,^11^. As deep learning has developed, convolutional neural networks (CNN) and deep-belief networks (DBN) have been used with increasing frequency for image classification. Compared with conventional machine-learning technologies (such as SVM and ANN), deep learning technology allows the input of original data and achieves a higher accuracy rate for classification^12^. Computer vision using deep learning technologies is widely used for object location, signal recognition and human face recognition^13^,^14^,^15^. In contrast, the application of deep learning technology to agricultural processes and area detection is still in its infancy.

Currently, research into the automatic and rapid detection of moulded grain is focused on the electronic nose, hyperspectral images and near-infrared spectrum technologies^16^,^17^,^18^,^19^. Although these technologies are effective and non-destructive testing methods, they still have some disadvantages compared to computer vision, such as being time-consuming and having lower locating abilities. Therefore, further study of computer vision methods to recognize the main infecting mould species and the mould colony positions on mouldy unhulled paddy is required.

This study investigates the potential of using computer vision with conventional and deep learning technologies to recognize both the infecting mould species and the mould colony positions on mouldy unhulled paddy. The specific aims of this study are (1) to build an SVM, a back-propagation neural network (BPNN) and a DBN model that can be used to classify images of unhulled paddy infected with different moulds; (2) to provide a pitch-segmentation recognition method combined with pitch-classification models using SVM, BPNN, CNN and DBN to recognize the mould colony areas in images of mouldy unhulled paddy; and (3) to compare the classification accuracy rates and speeds of the different classification models and thereby select the best methods to classify the infecting mould species and recognize the mould colony areas on mouldy unhulled paddy from the images.

## Results

### Characteristic parameter selection using the successive projection algorithm

**Figure 1:**
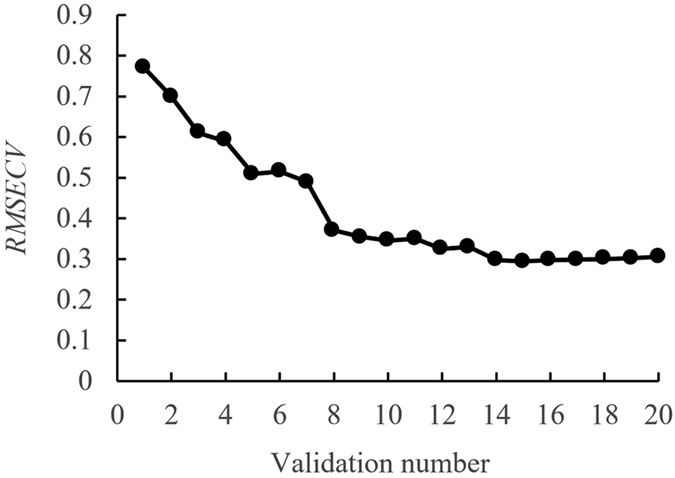
*RMSECV* of model cross validation as the validation number changes, when using SPA to reduce the original colour parameters of the sample image.

First, 64-D colour parameters that were extracted from mouldy unhulled paddy sample images were dimension-reduced using the successive projection algorithm (SPA). The number of selected variables was set from 1 to 20. The root mean square errors of cross validation (*RMSECV*) of the model cross variables, as calculated using different numbers of variables, are shown in Fig. 1. The *RMSECV* of the model cross variables decreased when the number of variables was decreased from 1 to 14 and remained stable when the number of variables was greater than 14. Therefore, fourteen variables were selected to replace the 64 variables of the original data. The 14 variables were *H*_13_, *R*_1_, *R*_9_, *R*_11_, *R*_13_, *G*_11_, *G*_12_, *B*_2_, *B*_3_, *B*_4_, *B*_5_, *B*_7_, *B*_8_ and *B*_9_, and *RMSECV* was 0.2977.

### Recognition of infecting mould species

#### Parameters and structures of models

**Figure 2:**
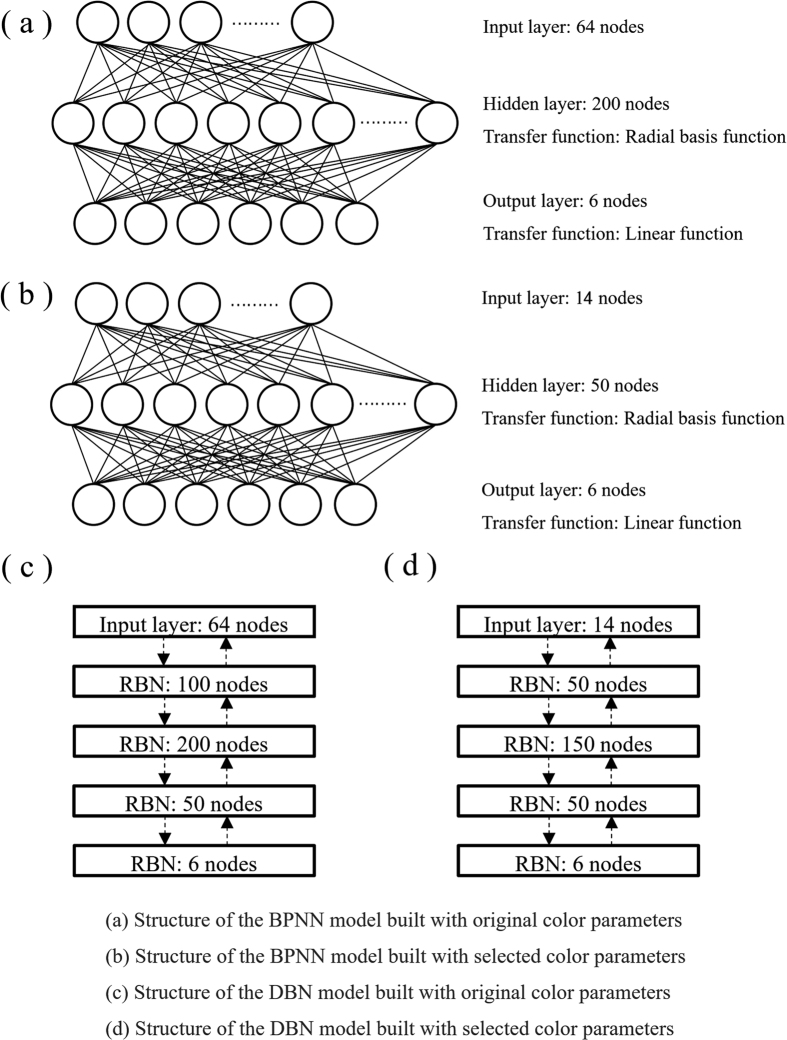
Structures of the BPNN and DBN models built with the original and the selected colour parameters of a sample image to recognize the infecting mould species.

The original 64-D colour parameters and the selected 14-D colour parameters were used to build both the conventional classification models of SVM and BPNN and the deep learning classification model of DBN. Due to the differences in the types of the models and the dimensions of the input data, different model parameters and structures were used when building the models. For the SVM models built with the original colour parameters and the selected colour parameters, a radial basis function was used as the kernel function, with a gamma value of 3 × 10^−9^ and a cost value of 100. The structures of the BPNN and DBN models built with the original colour parameters and the selected colour parameters are shown in Fig. 2. The BPNN model built with the original colour parameters has three layers: an input layer, a hidden layer and an output layer. The number of nodes in each layer was 64, 200 and 6, respectively. The transfer function of the first hidden layer was a radial basis function, and the transfer function of the second hidden layer was a linear function. The BPNN model built with the selected colour parameters was similar to the BPNN model built with the original colour parameters, except the number of nodes in each layer was 14, 50 and 6, respectively. The DBN model built with the original colour parameters had an input layer of 64 nodes and four layers of two-way junction restricted Boltzmann machines (RBNs). The number of nodes for each RBN was 100, 200, 50 and 6. The DBN model built with the selected colour parameters also had an input layer of 64 nodes and four RBN layers, but the number of nodes for each RBN was 50, 150, 50 and 6. The BPNN models showed stable classification ability after 500 training epochs, and the DBN models showed stable classification ability after 100 pre-training epochs for each RBN layer and 500 training epochs.

#### Classification accuracy rates of models

The classification accuracy rates of the training and the testing sets of sample images using the SVM, BPNN and DBN models built with the original and the selected colour parameters of each type of sample image are shown in Table 1. The classification accuracy rates of the training set sample images using all of the conventional and deep learning models built with the original and the selected colour parameters reached 100%. For the testing set sample image classification, the DBN model had the highest accuracy rate of the three types of models (99.4% without SPA; 100% with SPA), followed by the SVM model (98.9% without SPA; 99.4% with SPA) and the BPNN model (83.9% without SPA; 95% with SPA). The classification accuracy rates of the three types of models built with the selected colour parameters were higher than those of the same models built with the original colour parameters.

The results indicate that SPA is suitable for reducing the dimensions of the colour parameters of the sample images and that the DBN model achieved a higher accuracy rate than did conventional machine-learning models for building recognition models for infecting mould species. Pattern recognition for computer vision is effectively a black box method, so the signal-to-noise ratio (SNR) has a key effect on the recognition results. SPA can increase the SNR by reducing the amount of redundant data in the original data, thereby increasing the training and the testing speeds of the model by reducing the number of input variables. As a deep learning model, the DBN model is composed of multiple layers of RBNs, and each RBN layer is trained individually using the output data of the layer above and the feedback errors during the pre-training process, which makes the multilayer structure model reliable and easily trained. Compared with the DBN model, the BPNN model has a lower functional approximation ability because the error propagation method limits the number of effective layers (to no more than 3 layers). Thus, the DBN model built with the selected colour parameters by SPA was the best at recognizing the five infecting mould species contained in the mouldy unhulled-paddy images.

### Classification of the condition of the mildew on pitches segmented from the sample images

#### Classification accuracy rates

**Figure 3:**
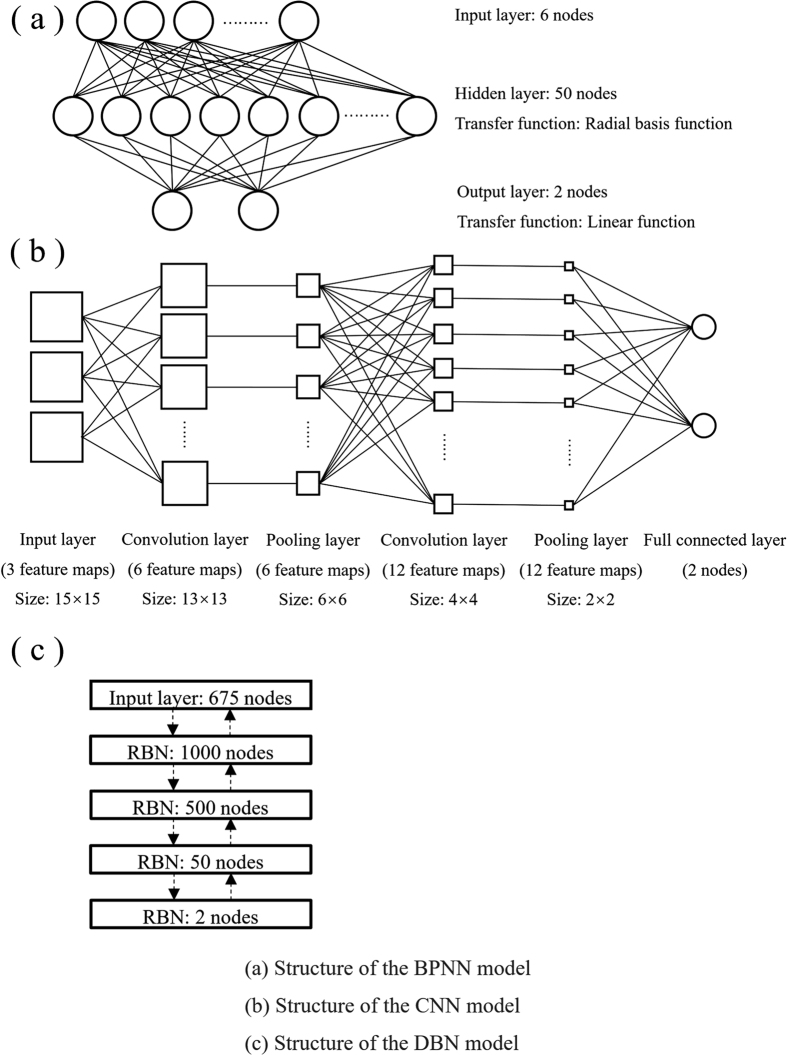
Structures of the BPNN, CNN and DBN models built for recognition of the mouldy condition of each pitch for each type of mouldy unhulled paddy image.

The SVM and BPNN models, which are typical conventional machine-learning models, were built and tested to classify pitches segmented from sample image into the target and the background pitches according to the pitch mildew conditions. A radial basis function was used as the kernel function for the constructed SVM model, with a cost value of 100 and a gamma value of 5 × 10^−5^ for the sample images with *Aspergillus nidulans* or *Aspergillus oryzae* or 5 × 10^−4^ for the sample images with *Aspergillus niger, Penicillum citrinum* or *Aspergillus versicolor*. The structure of the BPNN model built to classify the mildew conditions of pitches is shown in Fig. 3a. The BPNN model has three layers: an input layer, a hidden layer and an output layer. The number of nodes in each layer was 6, 50 and 2, respectively. The transfer function of the first hidden layer was a radial basis function, and the transfer function of the second hidden layer was a linear function.

The accuracy rates of the SVM and BPNN pitch-classification models of the training and testing set pitches segmented from the five types of sample images are shown in Table 2. For the SVM models, the accuracy rates for the pitch-classification from different types of sample images were over 90%, except for the classification of the pitches segmented from the sample images with *Aspergillus oryzae* (88.5%). However, for the BPNN model, only the accuracy rate for the classification of the pitches segmented from the sample images with *Aspergillus niger* was approximately 90%; those for the classification of the pitches segmented from other types of sample images were lower. There was also a large difference between the classification accuracy rates of the training and the testing set samples.

The CNN and DBN models, which are typical deep learning models, were built and tested to classify pitches segmented from sample image into the target and the background pitches according to the pitch mildew conditions. The structures of the CNN and DBN models are shown in Fig. 3b and c. The CNN model was composed of one input layer, two convolution layers, two pooling layers and one fully connected layer. The input layer had three feature maps, the first convolution layer and the first pooling layer each had six feature maps, and the second convolution layer and the second pooling layer each had twelve feature maps. The fully connected layer had two nodes for the result output. The size of the convolution kernel was 3 × 3. The DBN model was composed of one input layer of 675 nodes and four RBN layers. The number of nodes in each RBN layer was 1000, 500, 50 and 2.

The accuracy rates of the CNN and DBN pitch-classification models of the training and testing set pitches segmented from each type of sample image are shown in Table 2. For the CNN models, the accuracy rates for the classification of pitches from different types of sample images were over 90%, except for the classification of the pitches segmented from the sample images with *Aspergillus oryzae* (88.0%), and were similar to those of the SVM models. For the DBN models, the accuracy rates for the classification of pitches from the sample images with *Aspergillus nidulans, Aspergillus niger* and *Penicillum citrinum* were over 85%, but the accuracy rates for the classification of pitches segmented from the other two types of sample images were below 80%.

#### Recognition speed of the overall sample image with different classification models

The calculation time taken by the pitch segment recognition method combine with the different pitch-classification models to recognize the overall sample image was recorded. The calculation times of the pitch segment recognition method combine with the SVM, BPNN, CNN and DBN pitch-classification models were 139.6 ± 8.2 s, 213.3 ± 12.2 s, 26.2 ± 1.9 s and 51.5 ± 5.6 s, respectively.

Conventional machine-learning models take much longer to recognize a single sample image compared with the deep learning model. This is mainly due to the complex machine processing of characteristic parameter extraction for every pitch that is required in conventional machine-learning processes, which is unnecessary in deep learning classification. According to the accuracy rate and the calculation speed, of the four types of models, the CNN pitch-classification model is the best pitch-classification model for recognizing the mouldy condition of pitches segmented from mouldy unhulled paddy images.

#### Uniform CNN model for mildew condition classification of the pitches from all five types of sample images

**Figure 4:**
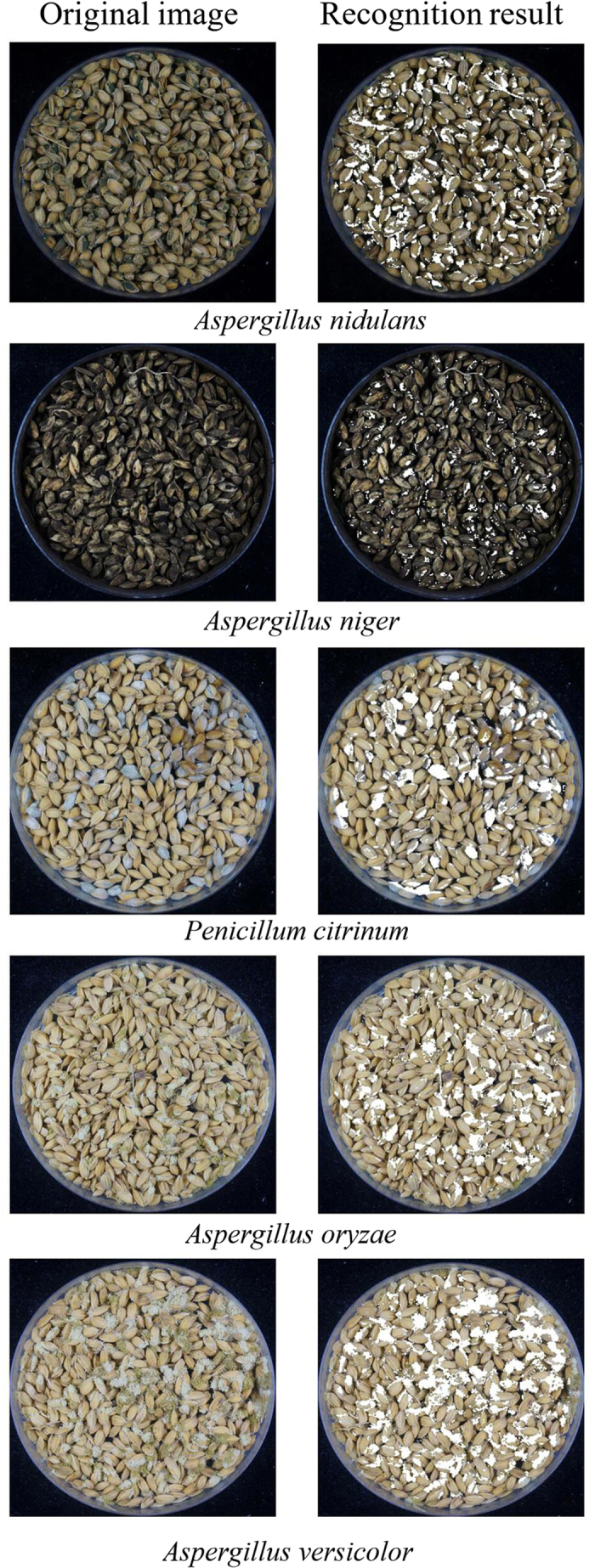
Recognition results of mould colony areas of the five types of sample images.

A uniform CNN model was built for the pitch classification of all five types of sample images. The accuracy rates of this CNN pitch-classification model of the training and testing sets for the five types of sample images are shown in Table 3. The average classification accuracy rate for pitches from all five types of sample images was 88.3% for the training set and 87.9% for the testing set. The lowest accuracy rates were obtained for the classification of pitches from sample images with *Aspergillus oryzae*, but the accuracy rates were still above 85%. The accuracy rates of the uniform CNN model for pitch classification were slightly lower than those of the independent CNN model for each type of sample image. The uniform CNN model was then used to recognize all of the sample images pre-processed pitch by pitch, and a binary image of the mould colony areas was obtained for each sample image. The recognition effects of the mould colony areas of the five types of sample image calculated with the uniform CNN model are shown in Fig. 4. Compared with the original sample images, the results show that the mould colony areas were accurately recognized.

## Discussion

Computer-vision technologies have been used to recognize mould colonies on unhulled paddy, specifically for mould species recognition and mould colony recognition and location. Compared to non-destructive testing technologies such as the electronic nose, hyperspectral images and near-infrared spectrum technologies for mouldy unhulled paddy^16^,^17^,^18^,^19^, this method is not suitable for storage monitoring because the early stages of mould growth are invisible, although some changes in odour and spectrum are already detectable by other methods. Otherwise, this method is suitable for mycotoxin reduction in mouldy unhulled paddy applications and for its mould colony location ability.

In the last two decades, much research has focused on rapid testing methods for agricultural products using computer vision and conventional machine learning technologies. Compared to conventional machine learning, deep learning technology has advantages in both classification ability and speed.

In this paper, the DBN model obtained better recognition results for infecting mould species in mouldy unhulled paddy compared with the SVM and BPNN models, probably due to its reliable multiple-layer structure composed of RBNs^20^. The SVM model can obtain good results for many different classification tasks in agricultural detection areas^21^,^22^,^23^. In this paper, the SVM model also demonstrated good performance for infecting mould species recognition, which was only slightly lower than that of the DBN model. However, the classification performances of SVM models depend on the support vectors that are extracted from the input data^24^. As the number of training samples increases, the number of support vectors in the SVM model also increases. When the number of training samples is large, the SVM will be complicated. The classification accuracy of BPNN for infecting mould species recognition with the selected data was approximately 11% higher than that with the original data, which indicates that BPNN is not stable for a large number of input variables.

Threshold segmentation technology has been used for image segmentation in almost all computer vision detection areas, especially in the detection of agricultural products using computer vision. However, it is difficult to segment areas of interest from an image with both complex colour and texture by using threshold segmentation. Mould colonies on unhulled paddies in an image have different colours and textures. Therefore, in this paper, pitch segmentation recognition methods combined with SVM, BPNN, CNN and DBN classification models were used to recognize the mould colony areas in the sample images, and the pitch segmentation recognition method combined with the CNN classification model was selected. The pitch segmentation recognition method has been used in remote-sensing images and medical image recognition^25^,^26^, which are also complicated image recognition tasks. For pitch classification, the CNN model applied in this paper is similar to the method used to classify satellite orthoimagery developed by Langkvist^27^, and the DBN recognition model used in this paper is similar to the method for segmenting and classifying epithelial and stromal regions in histopathological images developed by Xu^28^. According to the results of this paper, the CNN classification model was the best pitch classification model using the pitch segmentation recognition method due to its high classification speed and accuracy rate.

Deep learning techniques have also been used in hyperspectral imaging and speech recognition. Hu used the spectrum data of each pixel in a hyperspectral image and showed that the CNN model was also well able to classify 1-D array data^28^. Huang used the DBN and SVM models to recognize emotions in speech signals and showed that DBN had better performance but a longer training time^29^, which was similar to the results of this paper. The nodes in the DBN model were fully connected, and the parameters of each node could be affected by all of the nodes in the previous layer, whereas the convolution algorithm in the CNN model was only affected by nearby pixels^12^. This may be the reason for the lower accuracy rates of pitch recognition using the DBN model in this paper.

In this paper, the model training and testing codes were written in the Matlab language and run on a Matlab platform without multicore computing technology. The Matlab language is an interpreted language and thus has a much slower running speed than a compiler language, such as C and C++. A program for mouldy colony recognition on mouldy unhulled paddy images will be developed in the C or C++ language in future research to improve the recognition speed.

## Methods

### Preparation of mouldy unhulled paddy samples

#### Preparation of mould suspension

*Aspergillus nidulans, Aspergillus niger, Penicillum citrinum, Aspergillus oryzae* and *Aspergillus versicolor* were used as the infection moulds in this study. These mould strains were purchased from the Guangdong microbiology culture center and stored in a refrigerator at 4 °C. Of these mould strains, *Aspergillus nidulans, Penicillum citrinum* and *Aspergillus versicolor* are mycotoxin-producing moulds and can produce Sterigmatocystin and Citrinin. *Aspergillus niger* and *Aspergillus oryzae* are relatively safe.

The five mould strains were twice activated at 28 °C for three days before inoculation. Then, the mould cells of the five strains were washed in five test tubes to make the mould suspensions. A blood cell counting plate was used to detect the cell concentrations of the five mould suspensions. In each case, every measurement was replicated three times and an average value was determined. After that, every mould suspension was diluted to 10^6^ CFU/g using sterile water, according to the original mould cell concentration. The mould cell concentrations of the five mould suspensions before and after dilution are shown in Table 4.

#### Inoculation

The unhulled paddy used in this study was of the indica rice variety, bought from Jiangsu Sihong Farm and was stored in a refrigerator at 4 °C before inoculation. The unhulled paddy was placed in 120 culture dishes, with 15 g of unhulled paddy per culture dish. To eliminate the effect of the original microorganism on mould cultivation, the culture dishes filled with unhulled paddy were exposed to UV light for 30 minutes. Then, the five types of mould suspension that had been prepared were inoculated into the unhulled paddy in the culture dishes. Each type of mould suspension was added to 20 culture dishes, to which was added an additional volume of 3 mL per dish. The other 20 culture dishes that were filled with unhulled paddy were used as the control group, and 3 mL of sterile water was added. The inoculated unhulled-paddy samples were stored in an incubator (STIK instrument-equipment limited company) at a temperature of 25 °C and a relative humidity of 90%. Due to differences in the growth rates of the five species of mould, the samples inoculated with *Aspergillus niger, Aspergillus oryzae* and *Aspergillus versicolor* were stored for 42 days, and samples inoculated with *Aspergillus nidulans* and *Penicillum citrinum* were stored for 47 days. After storage, mould colonies were visible and were randomly distributed on the surfaces of all samples.

#### Sample-image capture

**Figure 5:**
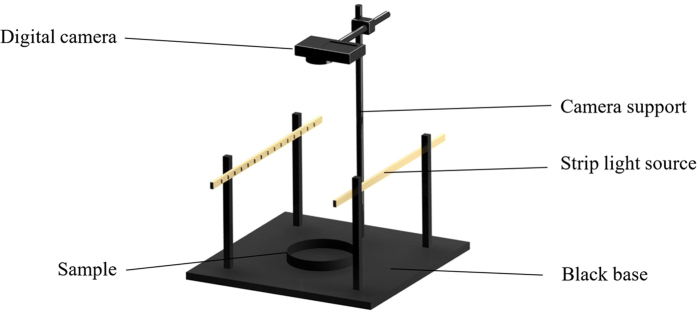
Self-made computer vision system.

To capture clear sample images of the mouldy unhulled paddy, a self-made computer vision system was used, as shown in Fig. 5. The self-made computer vision system contained a digital camera, two strip-light sources, a camera support and a black base. Each strip-light source was 33 cm long, and contained 12 white LED tamps. The power of each tamp was 1 W. The distances between the strip-light source and the base and between the strip-light sources were 15 cm and 20 cm, respectively. The digital camera used was a Sony Nex-6 digital camera, and the lens was a Sony SELP1650. The shooting parameters were a focal length of 30 cm and an exposure time of 1/15 s. The images were captured at a resolution of 4912 × 3264 and saved in .jpg format. To capture the mouldy unhulled paddy images, the strip-light sources were firstly turned on, and the culture dish filled with the mouldy unhulled paddy sample was placed on the base, directly facing the lens. Finally, the camera was triggered to capture the image.

Using this method, each mouldy unhulled paddy sample and control group sample was captured in five sample images at different angles. Thus, 100 sample images of each type of mouldy unhulled paddy sample and of a control group were obtained.

#### Image pre-processing

**Figure 6:**
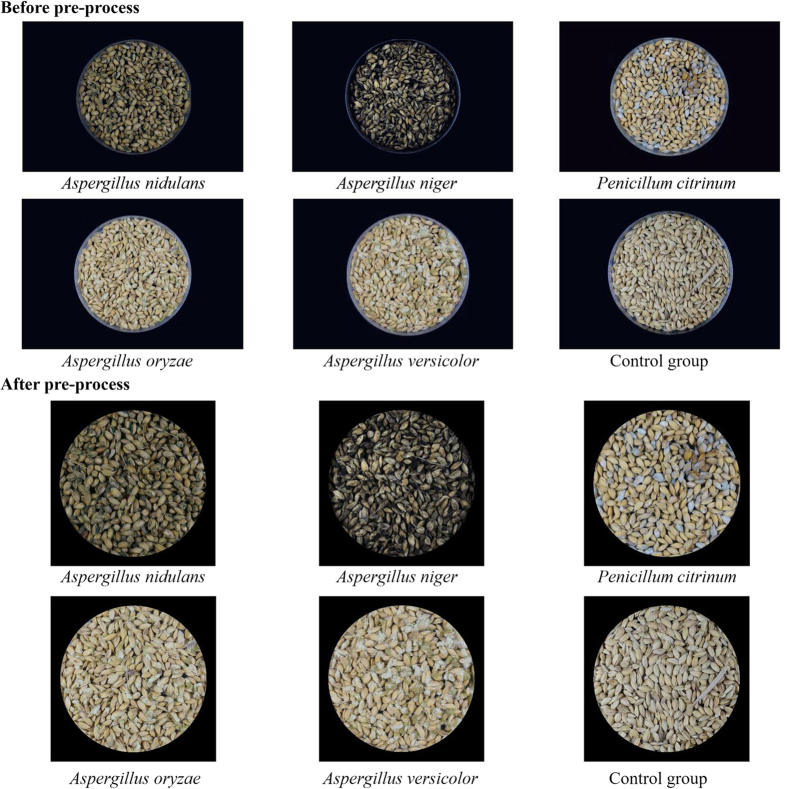
Images of mouldy unhulled paddy samples infected with five types of mould and the control group before and after image pre-processing.

The sample images were pre-processed using Matlab 2010b (Version 7.11, The Mathworks Inc., Natick, MA, USA, 2010). First, the sample images were segmented with a threshold grey value of 70 to obtain binary images of the culture dishes. Then, any redundant black background was removed, based on the largest external rectangle within the culture dish. Then, the resolution of each sample image was adjusted to 2490 × 2490, and the coordinates of the centre pixel were calculated. To eliminate the effect of the culture dish area, the pixel values were changed to zero if their distance to the centre pixel exceeded 1100 pixels. A selection of the sample images before and after pre-processing is shown in Fig. 6.

### Infecting mould species recognition method

#### Characteristic parameter extraction

**Figure 7:**
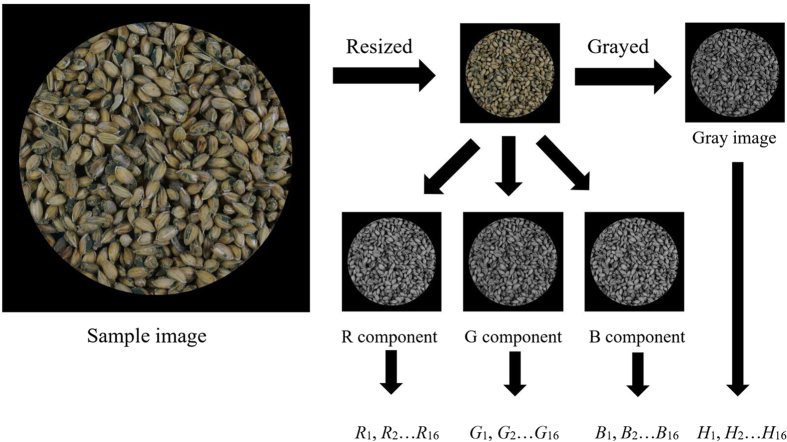
Characteristic parameters extraction process of colour information of a sample image.

Colour information is an important characteristic parameter that is used to recognize different objects both by humans and in computer vision. The colour information of mouldy unhulled paddy infection is different for different species and can be described by a colour histogram. Therefore, to recognize the infecting mould species, the colour-histogram parameters were extracted. The characteristic parameter extraction process is shown in Fig. 7. First, the resolution of the sample images was adjusted to 512 × 512, and the images were duplicated. One of the duplicates was converted to grey scale, and the other image was separated into three components (R, G and B). A 16-level grey-level histogram of the grey-scale image and the three colour-image components were then extracted, and the numbers of pixels within each tonal range were recorded as *H*_1_, *H*_2_ … *H*_16_ for the grey image, *R*_1_, *R*_2_ … *R*_16_ for the R component, *G*_1_, *G*_2_ … *G*_16_ for the G component, and *B*_1_, *B*_2_ … *B*_16_ for the B component.

#### Model building

The 64-D colour parameters extracted from the sample image using the image processing method may contain redundant and collinear data. To simplify the recognition model, the successive projection algorithm (SPA) was used to eliminate redundant and collinear data using Signal Processing and Variable Selection for Multivariate Calibration 1.0 software (developed by Araujo and Galvao)^29^,^30^,^31^.

The SVM and BPNN models are classical non-linear classification models that are based on conventional machine-learning techniques and are widely used in image classification research. The CNN and DBN models are classical classification models based on deep-learning techniques. The CNN model is designed to classify original image data without pre-processing but is not suitable for high-resolution images. BPNN is a basic model based on deep-learning techniques. DBN was developed from BPNN but has a larger hidden-layer structure and possesses functional approximation ability. CNN and DBN are effective classification models but have not been widely used, especially in agricultural detection. To develop an infecting mould species recognition method and compare the classification abilities of these models, the SVM, BPNN and DBN models were selected to classify the infecting mould species of mouldy unhulled paddy. The CNN model was not selected because the size of the sample images was too large.

Seventy sample images of each type of mouldy unhulled paddy and the control group were randomly selected as the training set, and the remaining 30 sample images were used as the testing set. Then, the 64 colour parameters of all sample images were extracted using the method described above for the training and testing sets. The 64 original colour-parameter data points and the colour-parameter data points selected using SPA were then used as the input data, and the species of the infecting mould in the sample images were used as the output data. SVM, BPNN and DBN models were built to recognize the species of the infecting mould in the sample images using the LabSVM 3.21 toolbox^32^, the Neural Network toolbox^33^ (The Mathworks Inc.) and the DeepLearnToolbox-master toolbox^34^ in Matlab 2010b.

### Mould colony area recognition in mouldy unhulled paddy

Mouldy areas in the sample images do not have a uniform colour, texture and shape, so the threshold segmentation method was useless for segmenting the mould colony areas in mouldy unhulled paddy. The pitch segmentation recognition method has the ability to recognize complex images, and proceeds as follows: first, the sample image is evenly segmented into small square pitches; then, some pitches are sampled to build a classification model to classify the target pitches and the background pitches; and finally, the target areas from the overall image are obtained by classifying all of the pitches segmented from the image using the previously built classification model. Considering the complexity of the images of mouldy unhulled paddy, a pitch segmentation recognition method combined with SVM, BPNN, DBN and CNN models was developed to recognize the mould colony area in mouldy unhulled paddy, and the best pitch classification model was selected.

#### Pitch segmentation

When segmenting pitches from the image, the size of each pitch segment should not be too large or small. Large pitch segments lead to a low resolution of the resulting binary image of the target area, and small pitch segments cause low pitch classification accuracy rates and long recognition times. The pitch size was determined to be 9 × 9 after multiple attempts, and the details of the procedure are as follows.

**Figure 8:**
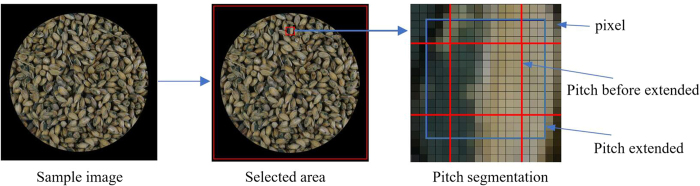
Image segmentation process.

As shown in Fig. 8, the pixel areas ranged from 4 to 2487 pixels in both the horizontal and vertical directions of the sample image, and they were segmented into 76176 (276 × 276) pitches on average, with a pitch resolution of 9 × 9 pixels. Then, the size of each pitch was extended to 15 × 15 pixels to make each pitch larger for easily classificating.

#### Pitch sample extraction

Ten sample images of unhulled paddy infected with each type of mould were randomly selected. Three small images of size 275 × 275 were then extracted from every selected sample image from the model-built images and segmented into 900 (30 × 30) pitch samples using the method described above. Thus, 27000 pitch samples were extracted for each type of sample image; 16000 of these samples were randomly selected as the training set, and the other 11000 pitch samples were used as the testing set.

#### Manual recognition of the pitch samples

**Figure 9:**
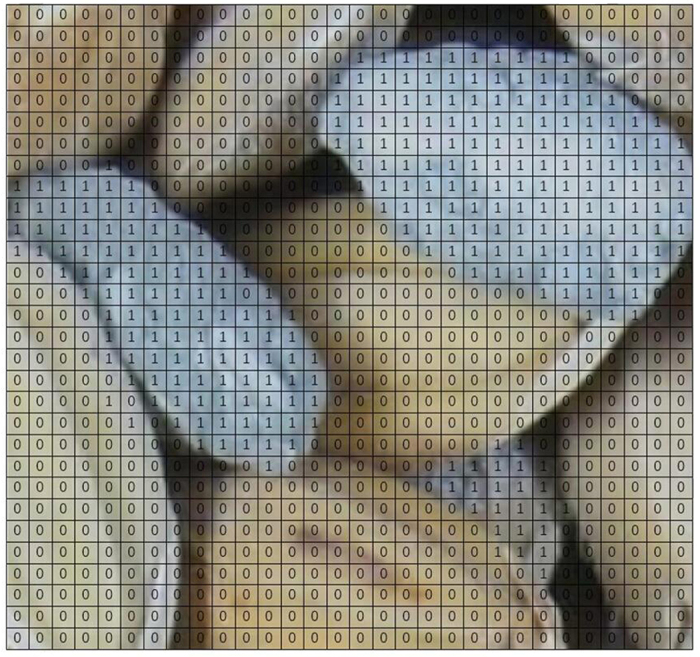
Pitch sample manual recognition using Microsoft Word 2016.

The model-built images taken from the pitch sample-extraction process were saved in jpg format and copied into the Microsoft Word 2016 software. A table with 30 rows, 30 columns and a uniform distribution of rows and columns was then created, and the table borders were aligned with the image borders. The image area in every table cell was taken as a pitch and manually recognized. As shown in Fig. 9, the number ‘1’ was typed into the table cell if more than 50% of the area of the pitch was filled with a visible mould colony, which indexed this pitch as a target pitch. Otherwise, the number ‘0’ was typed into the table cell, which indexed this pitch as a background pitch. The manual recognition results were used as the output data when building the classification models.

#### Mildew condition classification model built for the pitches from each type of sample image

To classify the pitches segmented from the image of mouldy unhulled paddy, compared the classification accuracies and speeds of the SVM, BPNN, CNN and DBN models, and select the best classification model to combine with the pitch segmentation recognition method to recognize the mould colony areas on the image, the SVM, BPNN, CNN and DBN classification models were used to classify the mildew condition of each pitch.

Conventional machine-learning technology requires characteristic parameter extraction for every pitch. Considering the amount of computation, simple colour and texture parameters were extracted from each pitch: *r, g* and *b* were the average grey values of the R, G and B components used as the colour parameters, and *stdr, stdg* and *stdb* were the standard deviation values of the R, G and B components used as the texture parameters. *r, g, b, stdr, stdg* and *stdb* of the pitches in the training and the testing sets of each type of sample image were calculated using Matlab 2010b. *r, g, b, stdr, stdg* and *stdb* were then used as the input data, and the pitch manual recognition results were used as the output data. The SVM and BPNN models use for the pitch recognition of each type of sample image were built using LabSVM 3.21 toolbox and Neural Network toolbox, respectively.

The R, G and B components of the pitch samples were used as the three input data matrices, and the manual recognition results were used as the output data. The CNN model for each type of sample image was built using the DeepLearnToolbox-master toolbox in Matlab 2010b with the input and output data of the pitch samples in the training set of each type of sample image and was tested using the pitch samples in the testing set.

The grey values of all pixels in each pitch were resized from a 15 × 15 × 3 data matrix to a 1-D data array with 675 elements. The data arrays of the sample pitches were used as the input data, and the manual recognition results were used as the output data. The DBN model for each type of sample image was built using DeepLearnToolbox-master toolbox in Matlab 2010b with the input and output data of pitch samples in the training set of each type of sample image and was tested using the pitch samples in the testing set.

#### Recognition speed test of the overall sample image with different classification models

The calculation speeds of two conventional machine-learning methods with the SVM and BPNN models and two deep learning methods with the CNN and DBN models were measured for the classification of all of the pitches that were segmented from one sample image. The hardware environment of this test was an Intel I7 4910 CPU with 16 G RAM, and the software environment was a Windows 7 system with the Matlab 2010b software. The calculation codes for the four methods, written using Matlab language, were almost the same, except the algorithms for the data classification each used a different model. The whole calculation process was as follows. The sample image was segmented into 76176 (276 × 276) pitches, and the 76176 pitches were recognized one by one using the previously built models. A result of 0 or 1 was obtained for every pitch, and these values were arranged according to the original pitch locations. Thus, a binary image of the mould colony area was created using the results of all pitches. The code for each recognition model was run five times, and the calculation times from the image segmentation to the binary image composition were recorded and averaged.

#### Uniform mildew condition classification model built for the pitches from all five types of sample images

The training and testing sets of the five types of sample images were combined into a new training set with 80000 pitches and a new testing set with 55000 pitches, respectively. The type of model for the pitch classification was selected from the four classification models (SVM, BPNN, CNN, and DBN) according to the results for the accuracy rates and calculation speeds in the previous test, and a new model was built using the pitch data in the new training set, and tested using the new testing set. Finally, the binary images for all of the sample images were calculated using the uniform mildew condition classification model by classifying all of the pitch segments from the sample images.

## Conclusions

The mould species recognition and mould colony recognition and location have been realized using a computer vision method with conventional and deep learning technologies. A recognition method of infecting mould species based on image processing and classification models (SVM, BPNN and DBN) and a pitch segmentation recognition method combined with SVM, BPNN, CNN and DBN classification models for mould colony area in mouldy unhulled paddy were developed. The DBN model for classifying different types of mouldy unhulled paddy images based on dimension-reduced colour-histogram parameters had the highest accuracy rate, at 100%, followed by the SVM and BPNN models. The pitch segmentation recognition method was used to recognize the mould colony areas on mouldy unhulled paddy. The accuracy rates with which the SVM and CNN models recognize the mildew conditions of the pitches segmented from each type of mouldy unhulled paddy images were approximately 90%, higher than those of the BPNN and the DBN models. The CNN and DBN models showed quicker (26.2 s and 51.5 s, respectively) calculation speeds when they were used to recognize all of the pitches in one sample image compared with the SVM and BPNN models (139.6 s and 213.3 s, respectively). A uniform CNN pitch classification model that is suitable for the five types of mouldy unhulled paddy sample images was built, and the classification accuracy was approximately 88% for both the training and the testing samples. The mould colony areas were easily found for each of the five types of mouldy unhulled paddy using the uniform CNN pitch classification model.

## Additional Information

**How to cite this article**: Sun, K. *et al*. Recognition of Mould Colony on Unhulled Paddy Based on Computer Vision using Conventional Machine-learning and Deep Learning Techniques. *Sci. Rep.* **6**, 37994; doi: 10.1038/srep37994 (2016).

**Publisher's note:** Springer Nature remains neutral with regard to jurisdictional claims in published maps and institutional affiliations.

## Figures and Tables

**Table 1:** Recognition accuracy rates of the training and the testing sets of the sample images using the SVM, BPNN and DBN models built with the original and the SPA-selected colour parameters.

Infecting mould species		SVM	SPA+ SVM	BPNN	SPA+ BPNN	DBN	SPA+ DBN
Training set	*Aspergillus nidulans*	100%	100%	100%	100%	100%	100%
	*Aspergillus niger*	100%	100%	100%	100%	100%	100%
	*Penicillum citrinum*	100%	100%	100%	100%	100%	100%
	*Aspergillus oryzae*	100%	100%	100%	100%	100%	100%
	*Aspergillus versicolor*	100%	100%	100%	100%	100%	100%
	Control group	100%	100%	100%	100%	100%	100%
	Average	100%	100%	100%	100%	100%	100%
Testing set	*Aspergillus nidulans*	100%	100%	63.3%	100%	100%	100%
	*Aspergillus niger*	100%	100%	83.3%	83.3%	100%	100%
	*Penicillum citrinum*	100%	100%	70%	100%	100%	100%
	*Aspergillus oryzae*	96.7%	100%	100%	100%	100%	100%
	*Aspergillus versicolor*	96.7	96.7%	96.7%	96.7%	96.7%	100%
	Control group	100%	100%	90%	90%	100%	100%
	Average	98.9%	99.4%	83.9%	95%	99.4%	100%

**Table 2:** Accuracy rates of the SVM, BPNN, CNN and DBN models in recognizing the mouldy conditions of the training and testing set pitches segmented from five types of sample images.

Infecting mould species	SVM		BPNN		CNN		DBN	
	Training	Testing	Training	Testing	Training	Testing	Training	Testing
*Aspergillus nidulans*	93%	92.4%	82.2%	74.5%	92.8%	92.3%	96.5%	90.8%
*Aspergillus niger*	92.5%	92.2%	89.7%	90.0%	93.4%	92.6%	95.8%	91.1%
*Penicillum citrinum*	92.1%	91.9%	79.8%	63.8%	91.0%	90.0%	88.9%	87.8%
*Aspergillus oryzae*	88.5%	88.3%	76.0%	85.1%	90.0%	88.0%	65.8%	65.1%
*Aspergillus versicolor*	92.0%	89.1%	80.2%	53.6%	91.3%	90.3%	58.3%	58.9%
Average	91.6%	90.8%	82.0%	73.4%	91.7%	90.6%	81.1%	78.7%

**Table 3:** Accuracy rates of the uniform CNN model to recognize the moldy condition of the training and testing sets pitches segmented from all of the five types of sample image.

Infecting mould species	Training	Testing
*Aspergillus nidulans*	89.8%	87.5%
*Aspergillus niger*	89.2%	89.6%
*Penicillum citrinum*	88.2%	88.2%
*Aspergillus oryzae*	88.4%	88.2%
*Aspergillus versicolor*	85.9%	86.0%
Average	88.3%	87.9%

**Table 4:** Concentrations of five mould suspensions before and after dilution.

Mould strain	Concentration before dilution (CFU/mL)	Concentration after dilution (CFU/mL)	Dilution ratio
*Aspergillus nidulans*	8 × 10^6^	1 × 10^6^	8
*Aspergillus niger*	4 × 10^6^	1 × 10^6^	4
*Penicillum citrinum*	8 × 10^6^	1 × 10^6^	8
*Aspergillus oryzae*	8 × 10^6^	1 × 10^6^	8
*Aspergillus versicolor*	12 × 10^6^	1 × 10^6^	12
